# Vesicoureteral Reflux in Children with Accidental Diagnosis of Unilateral Small Size Kidney

**DOI:** 10.5152/tud.2025.24060

**Published:** 2025-01-03

**Authors:** Mohsen Mohammadi, Khadijeh Ebrahimi, Soraya Khafri, Maryam Nikpour, Hadi Sorkhi

**Affiliations:** 1 Non-Communicable Pediatric Diseases Research Center, Health Research Institute, Babol University of Medical Sciences, Babol, I.R. Iran; 2 Student Research Committee, Health Research Institute, Babol University of Medical Sciences, Babol, IR Iran; 3 Social Determinants of Health Research Center, Health Research Institute, Babol University of Medical Sciences, Babol, I.R. Iran

**Keywords:** Children, vesicoureteral reflux, small-sized kidney, urinary tract infection

## Abstract

**Objective:**

A majority of small size kidney in children were diagnosed after a urinary tract infection (UTI) and with high-grade vesicoureteral reflux (VUR). This study was conducted in children who were diagnosed accidentally and investigated for VUR and UTI.

**Methods:**

This longitudinal retrospective study was conducted in children with a diagnosis of a small kidney accidentally discovered by ultrasonography and referred to Children’s Hospital in Babol, Iran, between 2012-2022. They were investigated by DMSA (dimercapto succinic acid) renoscintigraphy scans. Vesicoureteral reflux was diagnosed by voiding C\cystourethrography (VCUG) or radionuclide cystography (RNC). All children were followed for at least for 1 year with urine culture and urinalysis every 1-2 months for detection of UTI. Significance was set at *P* less than .05.

**Results:**

The mean age of the children with small-size kidneys was 5.52 ± 3.70 years, and 58.1% were boys. Out of the 86 children, 28 (32.6%) were found to have VUR, with approximately 71.4% of them being boys. Breaking down by gender, 40% of boys and 28.6% of girls with small-sized kidneys had VUR. Among the children with and without VUR, 42.9% and 10.3% experienced UTIs, respectively (*P* = .74). The predominant causative microorganism for UTIs was *Escherichia coli* (55.6%), with *Klebsiella* (22.4%) and *Enterobacter (22.4%)* accounting for the remaining cases.

**Conclusion:**

Accidental diagnoses of small-size kidneys in children revealed a notable presence of VUR, with a higher prevalence in boys. This suggests that VUR may constitute a significant etiological factor in the development of small-size kidneys. We recommend that these children must be evaluated for VUR.

## Introduction

The term “small kidney” refers to a reduction in kidney mass or volume, with varying definitions in the literature.^1^ In children, renal size varies with age and body size. The normal-sized kidney is defined as the distance between 1 to 4 lumbar vertebrae in 1.5-14-year-olds by the Currarino G study.^2^ The best noninvasive method for determining kidney size is ultrasonography.^3^ The definitive diagnosis of a small kidney relies on imaging techniques.^1^ Radiologic imaging may identify a small or hypoplastic kidney, with a 2 cm difference between kidneys^4^ or the largest longitudinal distance being less than 75% of the size difference.^5^ Differential function less than 45% on dimercapto succinic acid (DMSA) renoscintigraphy^6^ can be used for detection of a small-sized kidney.

While bilateral small kidneys are linked to decreased renal function and end-stage renal disease (ESRD),^7^ Children with unilateral small kidneys may exhibit normal serum creatinine levels due to compensatory function of the contralateral kidney.^8^ Causes of unilateral small kidneys include congenital hypoplasia, chronic damage from acute pyelonephritis, renal vascular accidents, irradiation, urological interventions, and high-grade vesicoureteral reflux (VUR).^9^ High-grade VUR, particularly, can lead to congenital renal damage before birth^10^ and contribute to kidney scars postnatally through urinary tract infections (UTIs), making UTI and VUR the most common causes of small size kidneys.^9b-11^ Moreover, posterior urethral valve could be a primary cause of hypoplastic kidneys in children.^7a^

Factors influencing the frequency of small kidneys include UTIs and other urological issues. In adults, it is reported that approximately 15.1% of patients with unilateral small kidneys were accidentally diagnosed.^11c^ Most diagnoses of small kidneys in children occur after UTIs and VUR.^9b,12^ However, the approach to small kidneys diagnosed incidentally after birth remains unknown, as there is a lack of studies addressing this specific scenario. This study aims to fill this knowledge gap by investigating children with incidentally diagnosed small kidneys. The participants were assessed for VUR and monitored for the recurrence of UTIs.

## Material and Methods

### Study Design and Sample

This retrospective cross-sectional study included all children under 18 years of age who were diagnosed with postnatal small-size kidneys and referred to Children’s Hospital in Babol, Iran, between 2012 and 2022. Inclusion criteria comprised children aged 2 months to 18 years, accidentally diagnosed with a small-size kidney via ultrasonography.^3^ Children with a history of(UTI or other urological conditions were excluded from the study. They were evaluated using ultrasonography for non-urology or nephrology complaints, and thus, small kidneys were diagnosed incidentally. This study was approved by the ethics committee of Babol University of Medical Sciences (Approval No: IR.MUBABOL.HRI.REC.1400.267, Date: 2022.2.20). Informed consent was obtained from the parents of all children.

### Data Collection

Children presenting with a diagnosis of a small-size kidney via ultrasonography were referred to the pediatric nephrology clinic of nephrology and subjected to DMSA renoscintigraphy scans. A kidney was deemed a small-size kidney if its uptake decreased by more than one-third or if more than 75% differential function was noted.^5^ Evaluation for VUR was conducted through voiding cystourethrography (VCUG) or radionuclide cystography (RNC). Antibiotic treatment with a prophylactic dose was administered to all children with VUR. The participants were monitored for at least 1 year, undergoing urinalysis and urine culture every 1-2 months for UTI detection. The grading of VUR was performed according to the international reflux study.^13^ Participants were categorized by age: less than 2 years, 2-5 years, and more than 5 years.

### Statistical Analysis

Data analysis utilized Statistical Package for Social Sciences (SPSS) version 22 (IBM SPSS Corp.; Armonk, NY, USA), and significance was set at *P* less than .05. Categorical and continuous variables were presented as counts (percentages) and mean ± standard deviation (SD), respectively.

## Results

Over the 10-year follow-up period, a total of 86 children with small-sized kidneys were included in the study. Among these, 50 (58.1%) were boys, while the remaining 41.9% were girls. The mean age of the children was 5.52 ± 3.70 years. Categorized by age, 3 (3.5%), 8 (9.3%), and 75 (87.2%) children were less than 2, 2-5, and more than 5 years old, respectively. Out of the 86 children with small-sized kidneys, 28 (32.6%) were found to have VUR, with approximately 71.4% of them being boys. Breaking down by gender, 40% of boys and 28.6% of girls with small-sized kidneys had VUR (Table 1). The distribution of VUR types revealed that 13 (46.4%) and 15 (53.6%) children had bilateral and unilateral VUR, respectively, resulting in a total of 41 units with VUR. According to VUR grading (Table 2), 4 (9.8%), 10 (24.4%), 14 (34.1%), 10 (24.4%), and 3 (7.3%) had VUR grades 1 to 5, respectively.

Among the children with VUR, 42.9% experienced UTIs, while in those without VUR, 10.3% had UTIs (*P* < .05). Out of 172 kidney units, 41 (28.8%) had VUR, with 27 of these having moderate to severe VUR (grades III-V) (Table 2). Stratifying by age groups, no VUR was observed in children under 2 years, but 12.5% and 36.8% of children in the 2-5 and more than 5 years groups had VUR, respectively. Multicystic dysplastic kidney and ureteropelvic junction obstruction were identified as causes of small kidneys in 13 (14.9%) and 12 (13.8%) patients, respectively. The predominant causative microorganism for UTIs was *Escherichia coli* (55.6%), with *Klebsiella*(22.4%) and *Enterobacter (22.4%)* accounting for the remaining cases. Throughout the follow-up period, 18 (20.1%) children experienced at least 1 episode of UTI, with 19.6% occurring in boys and 25% in girls (Table 1). No UTIs were recorded in children under 2 years, while 25% and 22.4% of children in the 2-5 and >5 years groups had UTIs, respectively. Statistical analysis did not reveal a significant difference between age (*P* = .74) groups and sex (*P* = .43) concerning UTIs.

## Discussion

The diagnosis and effective management of small kidneys have significant clinical importance, particularly in cases of bilateral small or hypoplastic kidneys where the risk of ESRD is heightened. Matsell et al^7a^’s study on children with bilateral hypoplastic kidneys indicated that the risk of ESRD is influenced by factors such as younger gestational age, kidney size at diagnosis, lower glomerular filtration rate, increased proteinuria, and higher blood pressure.

This study delves into the evaluation of VUR and the recurrence of UTIs in 86 children with small-size kidneys incidentally diagnosed at the Children’s Hospital in Babol, Iran. The findings reveal that 32.6% of children with incidentally diagnosed small-size kidneys had VUR. Unlike other studies where small kidneys were often diagnosed due to urological symptoms, such as loin pain, stone passage, or UTIs,^11c^ our study highlights the accidental diagnosis of small-size kidneys preceding the identification of VUR, a noteworthy observation given the relatively high frequency of VUR (about one-third) in these cases. In the general population the prevalence of VUR is about 0.4-2%, with most of them were diagnosed after UTI.^14^ In the Yilmaz S et al^15^ study, 19.4% of patients after first episode of UTI had renal scaring. Also Yilmas İ et al^16^ reported about 31% and 39% of children with febrile UTI and recurrent UTI had renal scaring. So, the diagnosis of scarring and then small kidneys (unilateral or bilateral) after UTI can be attributed to infection damage of kidney tissue. Thus small kidneys are expected.

Throughout the 1-year follow-up period in our study, 21.1% of patients experienced at least 1 episode of UTI. Given that UTIs are a primary cause of kidney damage, especially in conjunction with VUR.^7c,9b,10,17^ Our results underscore the significance of preventing UTIs in children with small-size kidneys and VUR.

The majority of our study participants exhibited moderate to severe grades of VUR, indicating a significant association between the grade of VUR and permanent kidney damage, subsequently leading to small-sized kidneys. Other studies also confirmed this issue.^10 11,18^ Interestingly, the history of patients before their small kidney diagnosis was largely unknown, with no reported instances of UTIs or other urinary tract issues. Although the use of antibiotic prophylaxis in children with VUR for the prevention of renal damage is^1,19 20^ doubtful, but our finding suggests that VUR and renal damage may have occurred either before delivery or as a consequence of UTIs after birth, emphasizing the need for comprehensive consideration of potential causes, including multicystic dysplastic kidneys and ureteropelvic junction obstruction.

Gender disparities were observed in the present study, with 58.1% of small kidney patients being boys, and 40% of them having VUR. In contrast, 36 (22.2%) girls with small kidneys had VUR. While literature on accidental diagnoses of small kidneys and subsequent VUR is scarce, studies focused on UTIs and VUR report varying results regarding gender involvement. Our study aligns with findings that suggest a higher risk of renal damage after VUR in boys.^7c,9b,10,12a,17,21^ About 2% of all pregnancies had urinary tract anomaly.^22^ Vesicoureteral reflux, ureteroplevic junction obstruction, and ureterovesical junction obstruction are the most common lower urinary tract anomaly.^23^ Kidney anomalies are diagnosed prenatally and is 20-30% of all urinary tract abnormalities.^23b,24^ Congenital anomalies of the kidney and urinary tract (CAKUT), renal agenesis, horseshoe kidney, and other urologic anomalies are more prevalent in males than females.^25^ So, similar to other reports, our study small kidneys were more in males than females.

Regrettably, the majority of patients (96.5%) were diagnosed with small kidneys after the age of 2, with 87.2% being over 5 years old. Of concern, 36.8% of patients over 5 years with small kidneys had VUR. Thus, heightened attention to VUR and UTIs in children older than 5 years with small kidneys is warranted. Our study was done on children with an accidental diagnosis of small size kidney. They were investigated for complaints such as abdominal pain. So, it is rational to find an accidental diagnosis of a small kidney in children more than 5 years old that can describe their complaints.

In this study, we followed the children for 1 year. Extending the follow-up period to 5-10 years would provide more comprehensive data on the progression of kidney disease and long-term outcomes. This was one of the limitations of the study, as well as the small sample size.

In conclusion, accidental diagnoses of small-size kidneys in children revealed a notable presence of VUR, with a higher prevalence in boys. This suggests that VUR may constitute a significant etiological factor in the development of small-size kidneys. Given the heightened risk of recurrent UTIs in these children, close follow-up and preventive measures against recurrent UTIs are imperative.

## Data Availability Statement:

The data of this study is available upon request to the corresponding author.

## Figures and Tables

**Table 1.** Frequency of UTI and VUR According to Age and Sex in Children with Small-Sized Kidney

Small Kidney Variable	VUR		UTI	
	Without VUR	With VUR	Without UTI	With UTI
	n (%)	n (%)	n (%)	n (%)
Boy	30 (60.0)	20 (40.0)	41 (82.0)	9 (18.0)
Girl	28 (77.8)	8 (28.6)	27 (75.0)	9 (25.0)
*P*-value	*P* ^a^ = .10		P^a^ = .43	
Age < 2 (Years)	3 (100)	–	3 (100)	–
Age 2–5 (Years)	7 (87.5)	1 (12.5)	6 (75.0)	2 (25.0)
Age > 5 (Years)	48 (63.2)	27 (36.8)	59 (77.6)	16 (22.4)
*P*-value	P^b^ = .17		P^b^ = .74	

^a^Chi-Score test

^b^Fischer’s exact test

UTI, urinary tract infection-; VUR, vesicoureteral reflux.

**Table 2.** Frequency of VUR According to Grading VUR in Children with Small-Sized Kidney

VUR N = 28	Grade	Left n (%)	Right n (%)	Total n (%)
	Grade1	2 (11.8)	2 (8.3)	4 (9.4)
	Grade 2	5 (29.4)	5 (20.8)	10 (24.4)
	Grade 3	4 (23.5)	10 (41.7)	14 (34.1)
	Grade 4	5 (24.4)	5 (20.8)	10 (24.4)
	Grade 5	1 (5.9)	2 (8.3)	3 (7.3)

VUR, vesicoureteral reflux
